# Intellectual capital, digital transformation and firms’ financial performance: Evidence from ecological protection and environmental governance industry in China

**DOI:** 10.1371/journal.pone.0316724

**Published:** 2025-01-27

**Authors:** Jian Yin, Jian Xu

**Affiliations:** School of Economics and Management, Qingdao Agricultural University, Qingdao, China; Bangladesh Institute of Governance and Management, BANGLADESH

## Abstract

As the pace of enterprise digital transformation accelerates, intellectual capital (IC) has become a core driving force of gaining market competitive advantages and enhancing value creation capabilities. The paper aims to investigate the impact of IC and its components on financial performance of Chinese ecological protection and environmental governance companies during 2018–2021. In addition, the moderating effect of digital transformation between them is examined. IC is measured by the modified value added intellectual coefficient (MVAIC) model, and the measurement of digital transformation is based on text mining. The results suggest that IC can improve firm financial performance, especially during COVID-19. Physical capital, human capital (HC), and relational capital (RC) positively affect financial performance, while structural and innovation capitals have no significant impact. In addition, digital transformation strengthens the positive relationship between IC and its two elements (HC and RC) and financial performance. Heterogeneous analysis finds that the relationship between RC and innovation capital and financial performance is positive before COVID-19, and it is not significant during COVID-19. For highly leveraged companies, structural capital negatively affects financial performance, and RC has a positive impact. These impacts are not significant for low leveraged companies. This paper provides some new insights for managers who seek new ways to improve firm performance in the process of digital transformation.

## 1. Introduction

As intellectual assets gradually become the main force of economic development, the traditional development model of enterprises has shifted from capital-driven to knowledge-driven [1]. The ascendancy of the knowledge economy has highlighted the significance of intellectual capital (IC). IC as an important intangible knowledge resource can help firms create value and bring them competitive advantages, which receives more and more attention from scholars [2–8]. Firms are viewed as a collection of multiple resources [9]. The resource-based view (RBV) emphasized that the utilization of resources is closely related to corporate long-term development. A company’s competitive advantage depends on the distinctive characteristics of internal resources that are difficult to duplicate [10]. Because of its scarcity, uniqueness, and difficulty in imitation, IC is regarded as an effective strategic resource for firms. Therefore, effective utilization of IC resources can improve firm performance. The current world is fraught with countless crises. The recent outbreak of the COVID-19 pandemic has posed a threat to the growth of the world economy [11], and organizations need to rely on IC to survive in turbulent times.

In addition, the COVID-19 crisis resulted in the emergence of a variety of digital business modes, which accelerates the digitalization progress in various industries [12]. There is no doubt that digital transformation has become an inevitable choice for Chinese companies to realize high-quality development [13]. Corporate digital transformation refers to the changes in production methods, organizational forms and other aspects triggered by the widespread application of digital technology, which can optimize corporate resources [14, 15]. Companies are experiencing digital transformation in the process of their operations. A successful digital transformation is not just process improvement and efficiency enhancement, and it can help companies achieve breakthroughs and bring new channels of creating value [16]. Existing studies have addressed the independent effect of IC and digital transformation on firm performance improvement, and scholars have neglected the combined effect of IC and digital transformation, which underscores a notable void in academic researches [17].

As global climate change intensifies and ecological and environmental issues become increasingly prominent, ecological protection and environmental governance have become a global consensus and an important issue for governments around the world. The rapid development of China’s economy has brought about serious ecological problems [18]. Ecological civilization and environmental governance has been raised to the national strategic level, and a series of environmental protection industry policies has provided a steady stream of power to the ecological protection and environmental governance industry. Its development is getting more and more attention, and it has become an important force of the industrial economic growth [19, 20]. This industry is highly knowledge-intensive and technology-driven [21], and the core competitiveness of companies in this industry is reflected in technological innovation and the accumulation of professional knowledge, all of which are important components of IC [22]. At present, research and development (R&D) level in China’s ecological protection and environmental governance sector is relatively low, and the relevant sectors are still in the early stages of development, which leads to the lack of efficient mechanisms for technology transfer [23]. In order to achieve green development, it is necessary to strengthen green technology innovation and the development of companies in this sector through effective IC utilization and reasonable digitalization. The development experience and achievements of China’s ecological protection and environmental governance industry have significant implications for other countries as valuable references.

This paper investigates the relationship between IC and its components and financial performance in China’s ecological protection and environmental governance sector during 2018–2021. It also examines whether the impact of IC and its elements on firms’ financial performance is different before and during COVID-19 and between highly leveraged companies and low leveraged companies. In addition, the moderating effect of digital transformation in this relationship is examined. The modified value added intellectual coefficient (MVAIC) model is used to measure IC, which quantifies the value created by IC in relation to traditional financial metrics. This model is widely utilized to assess the performance of IC in the current IC research [6]. Text analysis is used to measure corporate digital transformation, and multiple regression analysis is carried out for data analysis.

This paper contributes to the existing literature in the following aspects. First, it extends the relevant research by examining the impact of IC on firm financial performance in China’s ecological protection and environmental governance sector where little research has been done. In addition, this study examines whether the impact of IC and its elements on firms’ financial performance is different before and during COVID-19 and between highly leveraged companies and low leveraged companies. Second, it tries to uncover the moderating mechanism of digital transformation in the relationship between IC and firm performance. Finally, it will enable managers to have a deeper understanding of the role of digital transformation, and help them better improve value creation efficiency of IC resources in the digital transformation process.

The paper is structured as follows. Next section shows the literature review and hypotheses. Then, the methods are introduced followed by empirical results. The discussion is made based on these results. Finally, we conclude the paper.

## 2. Literature review and hypotheses development

### 2.1. IC and its composition

Since the 1990s, scholars have continued to define IC from different perspectives. Kaplan and Norton [24] defined IC as the accumulation of various intangible assets that a company invests in customers, suppliers, employees, and R&D. Edvinsson [25] defined it as the ability to improve organizational efficiency and gain a competitive advantage. Bontis [26] believed that IC includes patents, trademarks, brands, and other assets that cannot be reflected on the balance sheet. Although a unified definition has yet to be developed, it is certain that IC is an intangible asset that can create value [27, 28]. In this paper, IC is considered to be a complex and multidimensional concept that encompasses the non-material resources possessed by enterprises or individuals, such as knowledge, skills, experience, wisdom, innovative capabilities, and organizational relationships. These resources enable enterprises or individuals to create economic value and sustainable competitive advantage.

There are also different views among scholars about the composition of IC. Stewart [29] argued that IC consists of human capital (HC), structural capital (SC), and customer capital. Pulic [30] proposed that HC and SC are two IC elements. Ginesti et al. [31] pointed out that IC includes HC, organizational capital, and relational capital (RC). The current view widely accepted by scholars is that IC encompasses HC, SC, and RC [27, 28, 32]. HC refers to the knowledge, competencies, skills, and experience possessed by the employees of a firm [33]. SC can be regarded as the supporting infrastructure of HC, which includes factors such as processes, patents, databases, systems, and organizational culture and capabilities [34, 35]. RC refers to the various relationships with suppliers, partners, and customers [25, 34]. Some scholars [6, 36–39] pointed out that innovation capital should be added in the structure of IC components. In this study, HC, SC, RC, and innovation capital are considered to be the components of IC.

### 2.2. IC and firm financial performance

The RBV emphasized the uniqueness and heterogeneity of the resources owned by enterprises, and these resources are viewed as the source of competitive advantages. As an intangible asset, IC has uniqueness and difficulty in imitation. IC can form the unique competitiveness of an enterprise through the accumulation, dissemination, and application of knowledge. Moreover, the RBV underscored the effective allocation and utilization of resources. As a corporate strategic resource, IC requires sufficient attention and effective management. Enterprises should formulate appropriate strategies, optimize organizational structures, and enhance employee capabilities in order to fully leverage IC. Regarding HC, a high-quality workforce can improve production efficiency and product quality and reduce production costs, thus increasing sales revenue and profits. In addition, employees’ learning and innovation capabilities are also key factors driving technological progress and product upgrading, which can help enterprises explore new markets. With respect to SC, a well-structured organizational framework and management process can ensure the effective allocation of enterprise resources, reduce waste and redundancy, and improve overall operational efficiency. Concerning RC, establishing solid relationships with customers can improve their satisfaction and loyalty, which in turn leads to increased market share and sales revenue. Meanwhile, fostering strong partnerships with suppliers ensures the stability of the supply chain, reduces procurement costs and risks, and ultimately improves product quality and production efficiency. Pertaining to innovation capital, continuous technological innovation can enable enterprises to develop competitive new products and new technologies, thus increasing their market share and profitability.

Although the impact of IC dimensions on firm performance has been heatedly discussed by researchers worldwide, the results are still inconclusive. A large body of literature supports the view that IC is a driving force of firm performance. For example, Xu and Li [28] emphasized the importance of IC in improving firm performance. Zhang and Wang [40] found that investment in IC is positively related to corporate sustainable development. As per Pavlovic et al. [41], IC elements (HC and SC) improve financial performance in the fishing industry. The results of Zhang et al. [42] showed that physical capital and HC contribute to firm performance, while RC negatively influences firm profitability. Xu et al. [43] conducted a comparative analysis in two emerging markets and found that only human resources continue to enhance bank profitability during COVID-19. Nguyen et al. [44] used the value added intellectual coefficient (VAIC) model and found that IC, HC, SC and physical capital positively impact bank performance in Vietnam. Using structural equation modelling, Suharman et al. [45] reported a positive relationship between IC and the performance of state-owned companies. In the study of Barak and Sharma [46], IC, along with HC, SC, and RC has an impact on various performance indicators in Indian banking sector. Marten and Bui [47] found that IC and HC can promote the efficiency of banks in Vietnam. However, very few studies show conflicting results. Qomariah et al. [48] found that IC has no impact on the profitability of pharmaceutical firms during COVID-19. Kim and Tran [49] documented an insignificant relationship between HC and SC and business performance of Vietnamese small and medium enterprises. In addition, Liu and Kweh [50] and Xu and Zhang [51] confirmed the nonlinear relationship between IC and firm performance. Therefore, we come to the following set of hypotheses:

**Hypothesis 1a (H1a).** *IC improves financial performance of ecological protection and environmental governance companies*.
**Hypothesis 1b (H1b).** *HC improves financial performance of ecological protection and environmental governance companies*.
**Hypothesis 1c (H1c).** *SC improves financial performance of ecological protection and environmental governance companies*.
**Hypothesis 1d (H1d).** *RC improves financial performance of ecological protection and environmental governance companies*.
**Hypothesis 1e (H1e).** *Innovation capital improves financial performance of ecological protection and environmental governance companies*.

### 2.3. The moderating effect of digital transformation

Existing literature has largely focused on the economic impact of corporate digital transformation [52–54], and the role of digital transformation in the relationship between IC and firm performance is yet to be analyzed. According to the RBV, digital transformation can bring more valuable information resources, which is beneficial to the value creation efficiency of IC. By utilizing digital technologies such as big data and cloud computing, enterprises can delve deeper into the value behind data and discover new business opportunities [55], thus enhancing their market competitiveness and value creation capabilities. Next, an in-depth analysis will be conducted to explore how digital transformation affects the elements of IC.

HC is the most important component of IC. Corporate digital transformation improves the level of HC, which in turn enhances firm performance. On the one hand, digital transformation can optimize the structure of HC [56]. Although digital transformation can take away the routine jobs, it will lead to a rapid increase in the demand for knowledge-intensive employees. On the other hand, digital transformation promotes the fundamental changes in HC accumulation modes. Based on big data and artificial intelligence, the human-machine synergy not only replenishes the stock of HC, but also expands the knowledge of all employees. When high-quality HC engages in the production and operation process of enterprises, it will accelerate the transformation of knowledge [57].

Digital transformation makes organizational structure and management system more flexible [58], and the efficiency of SC is enhanced, thus stimulating firm performance. Digital transformation can drive the transformation of a company’s business model, operational model, and strategy [59]. It integrates internal and external resources through advanced technologies and reshapes corporate vision, strategy, organizational structure, processes, capabilities, and culture in order to adapt to the ever-changing digital world. In the process of digital transformation, companies can flexibly adjust the structure of functional departments according to the management needs. All these changes make corporate internal structure more resilient and flexible, which helps companies effectively deal with external environmental shocks.

The relationships with suppliers and customers are important components of an organization’s RC. Digital transformation can strengthen these relationships through enhancing information sharing and increasing the level of trust. On the one hand, digital transformation breaks down the boundaries between various links in the supply chain and lays the foundation for the interconnection of data and information among different entities. The supply chain ecosystem of open sharing and value co-creation has become the new form of enterprise-supplier-customer relationship in the era of digital economy [60]. Sharing information with suppliers can help optimize inventory management and promote suppliers’ participation in product design, thus achieving the goals of reducing raw material purchase costs and improving procurement efficiency. Exchanging and sharing information on product sales and market demand with customers can improve inventory turnover and reduce the negative impact of demand uncertainty. On the other hand, digital transformation enhances the level of trust between enterprises and suppliers and customers, thus reducing corporate default risks [61]. In the traditional situation, with the increase of supply chain participants, companies generally need to spend high costs to establish trust mechanisms with suppliers and customers. Based on the blockchain technology, the transaction records of supply chain participants will be permanently recorded and tracked at any time, which effectively prevents the default behavior of enterprises from the technical level. Long-term stable relationships with suppliers and customers can create competitive advantages for enterprises, thus increasing the value created by RC.

Innovation ability is important for any organization [62, 63]. The promotion effect of corporate digital transformation on innovation ability is mainly reflected in the three aspects. First, enterprise digital transformation reduces the uncertainty of innovation activities [64]. An important characteristic of innovation activities is high risky [65]. With the in-depth promotion of digital transformation, companies can accurately understand user demand through the application of digital technologies, reduce the uncertainty of innovation output, and promote product innovation from experience-driven to data-driven. In addition, digital design tools can accurately map physical entities into digital space, establish a real-time feedback mechanism, and greatly reduce the uncertainty of enterprise R&D. Second, digital transformation has changed the innovation mode of enterprises [66]. In the digital economy, enterprises can integrate a wider range of external innovation resources across organizational boundaries, so that the user or external R&D personnel can participate in the whole process of product value creation, achieving the open network of innovation. Third, digital transformation improves the management efficiency of innovation activities. Digital transformation changes the internal organizational structure from hierarchical to flat, which not only helps better grasp the external innovation opportunities, but also promotes the exchange and sharing of data and knowledge, thus enhancing companies’ innovation capability. The stronger the innovation ability, the more value innovation capital creates. Therefore, we come to the following set of hypotheses:

**Hypothesis 2a (H2a).** *Digital transformation has a moderating effect between IC and financial performance*.
**Hypothesis 2b (H2b).** *Digital transformation has a moderating effect between HC and financial performance*.
**Hypothesis 2c (H2c).** *Digital transformation has a moderating effect between SC and financial performance*.
**Hypothesis 2d (H2d).** *Digital transformation has a moderating effect between RC and financial performance*.
**Hypothesis 2e (H2e).** *Digital transformation has a moderating effect between innovation capital and financial performance*.

## 3. Methodology

### 3.1. Sample

The original sample includes ecological protection and environmental governance companies listed on the Shanghai, Shenzhen, and Beijing stock exchanges during 2018–2021. Since 2017, the Chinese government has consecutively included the development of digital economy in its government work reports and explicitly proposed in the 14th Five-Year Plan to drive the transformation of production, lifestyle and governance methods through digital transformation, which accelerates the process of digital transformation at both the company and national levels. Therefore, the year 2018 is selected as the starting point. Companies with missing information, delisted companies, and special treatment (ST) companies are deleted from our research. Table 1 shows the detailed information about sample distribution. Finally, 60 firms with 171 observations are left in this study. Data are collected from the China Stock Market & Accounting Research Database (CSMAR) database. The CSMAR database is a research-oriented database in the field of economics and finance developed in accordance with China’s actual national conditions. Stata 17.0 is used to analyze the data.

**Table 1:** Sample distribution.

Original company sample	81
Companies with missing information	16
Delisted companies	1
Special treatment (ST) companies	4
Final company sample	60
Total firm-year observations	171

### 3.2. Variables

**Fig 1:**
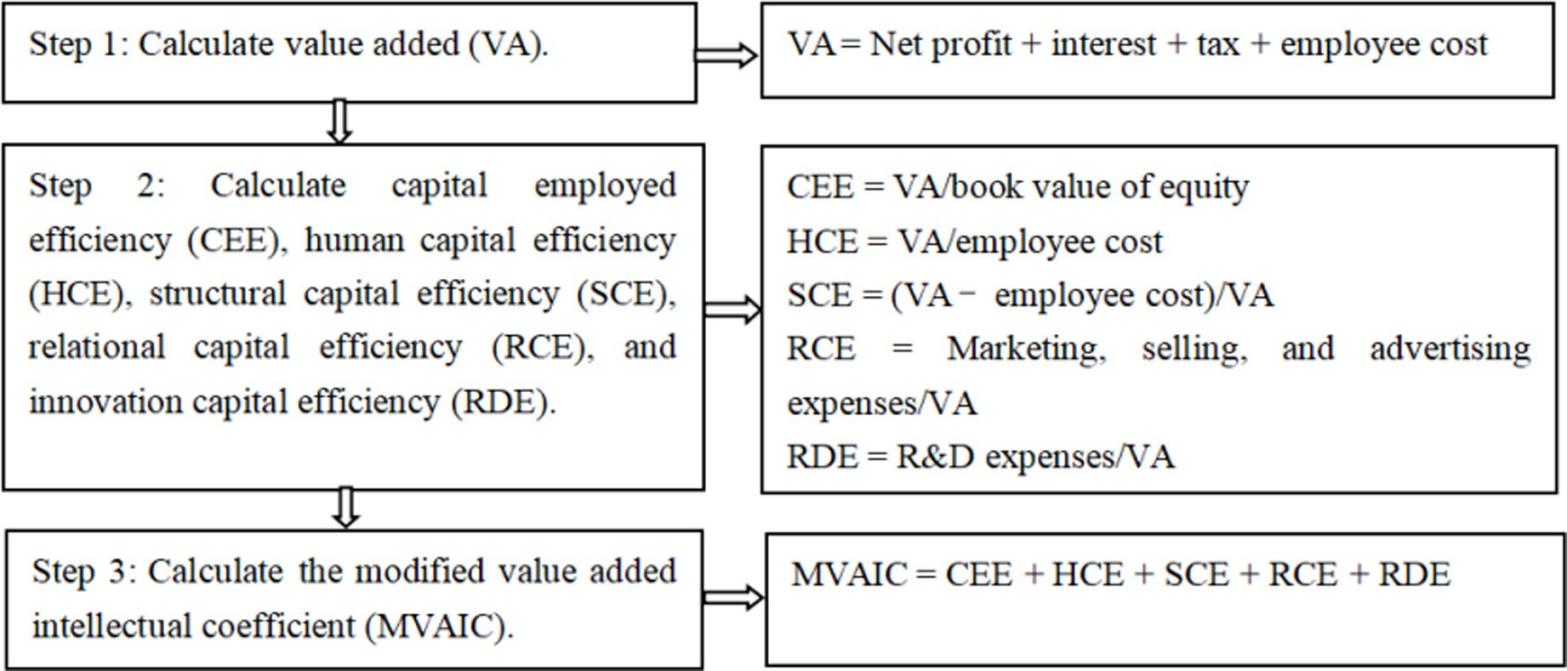
The calculation processes of IC.

1. Dependent variables. This study uses return on assets (ROA) to measure firm financial performance. It is an accounting-based indicator that is widely used in academics [5, 6, 28, 36, 39, 41, 43, 51, 67]. In order to ensure the robustness of our study, return on equity (ROE) is used as an alternative.
2. Independent variables. How to accurately assess IC has become an urgent task for practitioners [28]. Guided by Xu et al. [6], Xu and Zhang [39], Xu and Liu [68], and Ge and Xu [69], the MVAIC model is used to assess IC. Fig 1 displays the calculation processes of IC.

3. Moderator variable. Gudied by Liu et al. [13], Wu et al. [70], and Du et al. [71], this study uses text analysis to measure the level of digital transformation. It is measured by the natural logarithm of the number of keywords plus one.
4. Control variables. Referring to previous literature [6, 13, 28, 36, 39, 42, 43, 50, 68, 69, 72, 73], this study chooses firm scale (SIZE), debt ratio (LEV), firm ownership (OWN), firm age (AGE), sales growth rate (SALES) as control variables. SIZE is expected to have a positive impact on financial performance [28, 36, 73]. Companies with more liabilities face more financial risks and have relatively poorer performance [28, 36, 39, 73]. Private companies generally have better performance than state-owned companies [67]. Mature companies have sufficient funds and resources, which leads to high performance [6]. A high sales growth rate is often a sign of financial health to a company [28, 73]. In addition, year-fixed effect (YEAR) is included in models. The specific measurements of all variables are listed in Table 2.

**Table 2:** Variable definition.

Variable	Symbol	Measurement
Return on assets	ROA	Net income/average total assets
Return on equity	ROE	Net income/average shareholders’ equity
Modified value added intellectual coefficient	MVAIC	CEE + HCE + SCE + RCE + RDE
Capital employed efficiency	CEE	VA/book value of equity
Human capital efficiency	HCE	VA/employee cost
Structural capital efficiency	SCE	(VA–employee cost)/VA
Relational capital efficiency	RCE	Marketing, selling, and advertising expenses/VA
Innovation capital efficiency	RDE	R&D expenses/VA
Digital transformation	DIGITAL	Natural logarithm of the number of keywords plus 1
Firm scale	SIZE	Natural logarithm of total assets
Debt ratio	LEV	Total liabilities/total assets
Firm ownership	OWN	Dummy variable that takes 1 if a company is state-owned, 0 otherwise
Firm age	AGE	Natural logarithm of years since corporate setup
Sales growth rate	SALES	(Current year’s sales–last year’s sales)/last year’s sales
Year	YEAR	Dummy variable that takes 1 for the test year, 0 otherwise

### 3.3. Models

This study uses the following model to examine the impact of IC and its elements on the ROA indicator.


$$ROA_{i,t}={ß}_{0}+{ß}_{1}X_{i,t}+{ß}_{2}SIZE_{i,t}+{ß}_{3}LEV_{i,t}+{ß}_{4}OWN_{i,t}+{ß}_{5}AGE_{i,t}+{ß}_{6}SALES_{i,t}+\sum YEAR+\varepsilon_{i,t}$$ (1)

This study uses the following model to examine the moderating effect of digital transformation.

$$ROA_{i,t}={ß}_{0}+{ß}_{1}DIGITAL_{i,t}+{ß}_{2}X_{i,t}+{ß}_{3}X_{i,t}\times DIGITAL_{i,t}+{ß}_{4}SIZE_{i,t}+{ß}_{5}LEV_{i,t}+{ß}_{6}OWN_{i,t}+{ß}_{7}AGE_{i,t}+{ß}_{8}SALES_{i,t}+\sum YEAR+\varepsilon_{i,t}$$ (2)

where X includes (1) MVAIC, (2) CEE, (3) HCE, (4) SCE, (5) RCE, and (6) RDE; i represents firm; t represents the year; ß is the parameters; ε is the term error.

## 4. Results

### 4.1. Descriptive statistics

Table 3 shows the descriptive statistics. The mean ROA (0.0374) suggests that the profitability of sampled companies is low. In our sample, about 90 percent of these companies can earn profits. MVAIC has a mean value of 3.8242, which means that an investment of one monetary unit yields 3.8242 monetary units. Among IC components, HC has the greatest value creation efficiency followed by SC and innovation capital. The sum of mean values of HCE, SCE, RCE, and RDE is much greater than that of CEE, indicating that intangibles have a dominant role in the process of value creation. DIGITAL has a mean value of 0.9723 with a maximum of 4.2047 and a minimum of 0, which implies that the level of digital transformation varies greatly. SIZE has a mean value of 22.3296, and LEV has a mean value of 0.5096, reflecting that such companies have good capital structure. About 28 percent are state-owned companies. The mean value of SALES is 0.1942.

**Table 3:** Descriptive statistics.

Variable	N	Mean	Max	Min	SD
ROA	171	0.0374	0.2000	-0.3046	0.0632
MVAIC	171	3.8242	12.1237	-9.1998	2.4377
CEE	171	0.1764	0.9034	-2.2246	0.2410
HCE	171	2.7942	11.0059	-8.0129	2.2610
SCE	171	0.6433	9.7302	-4.0344	1.1561
RCE	171	0.0726	1.1895	-2.1956	0.2715
RDE	171	0.1377	3.3734	-2.4558	0.4074
DIGITAL	171	0.9723	4.2047	0	1.0264
SIZE	171	22.3296	24.9836	19.5312	1.1968
LEV	171	0.5096	0.8702	0.0570	0.1842
OWN	171	0.2807	1	0	0.4507
AGE	171	2.8886	3.6109	1.9459	0.3347
SALES	171	0.1942	9.0640	-0.6825	0.7612

### 4.2. Correlation analysis

The results of correlation analysis are shown in Table 4. As shown in Table 4, MVAIC, CEE, HCE, RCE are positively related to ROA, while DIGITAL is negatively associated with ROA. SCE and RDE is not associated with ROA. When calculating the values of variance inflation factor (VIF), all of them are less than 4, suggesting that multi-collinearity is not serious in this study.

**Table 4:** Correlation matrix.

Variable	1	2	3	4	5	6	7	8	9	10	11	12	13
1 ROA	1												
2 MVAIC	0.694*** (12.51)	1											
3 CEE	0.659*** (11.40)	0.517*** (7.84)	1										
4 HCE	0.703*** (12.85)	0.971*** (52.87)	0.494*** (7.38)	1									
5 SCE	-0.091 (-1.18)	0.208*** (2.76)	-0.118 (-1.53)	-0.001 (-0.02)	1								
6 RCE	0.154** (2.02)	-0.149* (-1.91)	0.098 (1.28)	0.036 (0.47)	-0.947*** (-8.94)	1							
7 RDE	0.014 (0.18)	-0.201** (-2.57)	0.030 (0.39)	-0.052 (-0.67)	-0.887*** (-8.63)	0.871*** (23.08)	1						
8 DIGITAL	-0.202*** (-2.57)	-0.245*** (-3.10)	-0.022 (-0.29)	-0.237*** (-3.00)	-0.068 (-0.89)	0.040 (0.53)	0.028 (0.36)	1					
9 SIZE	-0.223*** (-2.83)	0.038 (0.50)	0.099 (1.29)	0.073 (0.95)	-0.072 (-0.94)	-0.036 (-0.47)	-0.007 (-0.10)	0.092 (1.21)	1				
10 LEV	-0.494*** (-5.75)	-0.147* (-1.89)	-0.049 (-0.64)	-0.140* (-1.80)	0.006 (0.08)	-0.090 (-1.16)	-0.032 (-0.41)	0.132* (1.73)	0.663*** (11.50)	1			
11 OWN	-0.150* (-1.932)	-0.179** (-2.30)	0.031 (0.41)	-0.151* (-1.94)	-0.119 (-1.53)	0.014 (0.18)	0.073 (0.95)	0.097 (1.26)	0.495*** (7.41)	0.428*** (6.16)	1		
12 AGE	-0.266*** (-3.34)	-0.247*** (-3.12)	-0.003 (-0.04)	-0.238*** (-3.00)	-0.101 (-1.31)	0.080 (1.04)	0.074 (0.97)	0.200*** (2.66)	0.420*** (6.01)	0.255*** (3.42)	0.092 (1.203)	1	
13 SALES	0.174** (2.30)	0.167** (2.20)	0.341*** (4.72)	0.173** (2.28)	-0.045 (-0.58)	0.009 (0.11)	-0.040 (-0.52)	-0.038 (-0.49)	0.152** (2.00)	0.113 (1.48)	-0.060 (-0.77)	0.085 (1.11)	1

Notes: * *p* < 0.10

** *p* < 0.05

*** *p* < 0.01. *t*-values are in parentheses.

### 4.3. Empirical results

Table 5 presents the results of Models (1)–(6). In Model (1), the coefficient of MVAIC is positive and significant at the 1% level (ß = 0.016, t = 12.54). Therefore, H1a is fully supported. This supports the findings of previous research [5, 6, 28, 31, 36–39, 41–43, 68, 69]. Model (2) shows a significant positive relationship between CEE and ROA (ß = 0.158, t = 12.46), which suggests that physical assets have an impact on firm financial performance. In addition, the impact of physical assets is greatest, which suggests that such companies largely depend on fixed assets to maintain operation. Regarding IC elements, the coefficients of HCE and RCE are positive and statistically significant (ß = 0.018, t = 13.06; ß = 0.033, t = 2.25). RCE has a greater influence than HCE. It could be because that in this sector companies provide products or services to help their customers deal with pollutants. Therefore, H1b and H1d are fully supported. The coefficient of SCE in Model (4) is negative but insignificant (ß = -0.005, t = -1.53), which rejects H1c. In Model (6), the coefficient of RDE is positive but insignificant (ß = 0.006, t = 0.61). Therefore, H1e is not accepted. It might be explained by the fact that compared to developed countries, the level of R&D investment in this sector is very low, which hinders the independent innovation capability. Similarly, Gupta and Raman [74] reported that IC, HC, RC, and physical capital positively affect ROA, while innovation capital does not influence ROA.

**Table 5:** Regression results of Models (1)–(6).

Variable	Model (1)	Model (2)	Model (3)	Model (4)	Model (5)	Model (6)
	Independent variable: MVAIC	Independent variable: CEE	Independent variable: HCE	Independent variable: SCE	Independent variable: RCE	Independent variable: RDE
Constant	0.116* (1.67)	0.053 (0.77)	0.168** (2.46)	-0.001 (-0.01)	-0.015 (-0.16)	-0.015 (-0.15)
X	0.016** (12.54)	0.158** (12.46)	0.018** (13.06)	-0.005 (-1.53)	0.033** (2.25)	0.006 (0.61)
SIZE	-0.004 (-0.93)	0.006* (1.79)	-0.006 (-1.50)	0.012** (2.46)	0.013** (2.57)	0.013** (2.50)
LEV	-0.160** (-7.54)	-0.175** (-8.27)	-0.153** (-7.28)	-0.221** (-7.63)	-0.218** (-7.61)	-0.224** (-7.71)
OWN	0.028** (3.71)	0.0003 (0.04)	0.027** (3.64)	0.003 (0.31)	0.004 (0.35)	0.005 (0.44)
AGE	0.002 (0.16)	-0.035** (-3.76)	0.003 (0.35)	-0.043** (-3.30)	-0.044** (-3.42)	-0.042** (-3.20)
SALES	0.011** (2.91)	0.002 (0.37)	0.011** (2.88)	0.018** (3.39)	0.018** (3.46)	0.018** (3.46)
YEAR	Yes	Yes	Yes	Yes	Yes	Yes
N	171	171	171	171	171	171
Adj. R^2^	0.6874	0.6678	0.6832	0.3567	0.3673	0.3488
F	39.33**	38.98**	41.73**	11.47**	11.97**	11.12**

Notes: * *p* < 0.10

** *p* < 0.05

*** *p* < 0.01. *t*-values are in parentheses.

Table 6 shows the results of the moderating role of digital transformation between IC and its elements and financial performance. It should be noticed that the coefficients of DIGITAL are negative in Models (7)–(12), indicating that digital transformation is detrimental to firm financial performance. The increase in operating costs and administrative expenses might lead to the decline in firm performance in the process of digital transformation [75]. This is not consistent with many researchers [13, 15, 16, 60] who claimed that digital transformation brings great advantages to businesses. Hou et al. [76] and Zhao and Ren [77] concluded that digital transformation can promote technology innovation, and there is an inverted U-shaped relationship. In Model (7), the interaction MVAIC×DIGITAL is significantly positive (ß = 0.005, t = 4.69). Therefore, H2a is supported. Similarly, the positive coefficients of HCE×DIGITAL and RCE×DIGITAL lead to the support of H2b and H2d. Digital technology can help enterprises achieve precision marketing and personalized services and improve customer satisfaction and loyalty. In addition, the coefficients of SCE×DIGITAL and RDE×DIGITAL are not significant at the 5% level. Therefore, H2c and H2e are not supported. The coefficient of CEE×DIGITAL is positive and significant (ß = 0.035, t = 2.93), which means digital transformation positively moderates the impact of physical assets on firm financial performance.

**Table 6:** Regression results of Models (7)–(12).

Variable	Model (7)	Model (8)	Model (9)	Model (10)	Model (11)	Model (12)
	Independent variable: MVAIC	Independent variable: CEE	Independent variable: HCE	Independent variable: SCE	Independent variable: RCE	Independent variable: RDE
Constant	0.146** (2.22)	0.083 (1.24)	0.199*** (3.12)	0.002 (0.02)	-0.018 (-0.19)	-0.014 (-0.15)
DIGITAL	-0.018*** (-3.74)	-0.011*** (-3.38)	-0.014*** (-3.77)	-0.003 (-0.53)	-0.009** (-2.07)	-0.009* (-1.93)
X	0.013*** (8.60)	0.137*** (9.60)	0.014*** (9.14)	-0.003 (-0.69)	0.017 (0.94)	-0.004 (-0.32)
X×DIGITAL	0.005*** (4.69)	0.035*** (2.93)	0.006*** (5.28)	-0.005 (-0.90)	0.043* (1.69)	0.022 (1.59)
SIZE	-0.005 (-1.31)	0.005 (1.50)	-0.007** (-2.01)	0.012** (2.36)	0.013** (2.56)	0.012** (2.44)
LEV	-0.150*** (-7.43)	-0.171*** (-8.34)	-0.142*** (-7.22)	-0.218*** (-7.50)	-0.216*** (-7.53)	-0.223*** (-7.67)
OWN	0.030*** (4.21)	0.003 (0.35)	0.029*** (4.24)	0.004 (0.34)	0.004 (0.39)	0.004 (0.39)
AGE	0.004 (0.44)	-0.034*** (-3.65)	0.006 (0.71)	-0.040*** (-2.99)	-0.040*** (-3.05)	-0.037*** (-2.79)
SALES	0.011*** (3.08)	-0.002 (-0.38)	0.011*** (3.17)	0.018*** (3.35)	0.019*** (3.57)	0.019*** (3.55)
YEAR	Yes	Yes	Yes	Yes	Yes	Yes
N	171	171	171	171	171	171
Adj. R^2^	0.7064	0.6899	0.7271	0.3599	0.3784	0.3581
F	38.19***	35.39***	42.18***	9.69***	10.41***	9.62***

Notes: * *p* < 0.10

** *p* < 0.05

*** *p* < 0.01. *t*-values are in parentheses.

As for control variables, it is obvious that LEV and AGE negatively influence the ROA indicator, whereas SALES has a positive impact on ROA. In addition, this study uses ROE as an alternative for ROA and re-estimate Models (1)–(12). The results of robustness check are similar to the previous results in Tables 5 and 6. Therefore, our results are robust.

### 4.4. Heterogeneous analysis

This study examines whether the impact of IC and its elements on firms’ financial performance is different before and during COVID-19. The results are displayed in Tables 7 and 8. Tables 7 and 8 show that IC, physical capital, and HC positively affect ROA before and during COVID-19, suggesting that companies still need to attach importance to the investment in IC, especially HC. SC has no significant impact on ROA before and during COVID-19 pandemic. It is noticing that RC and innovation capital exert a significant and positive influence on the ROA indicator before COVID-19, while they have no impact after the outbreak of COVID-19. This could be due to the disruption of supply chains caused by COVID-19.

**Table 7:** Regression results of Models (1)–(6) before COVID-19.

Variable	Model (1)	Model (2)	Model (3)	Model (4)	Model (5)	Model (6)
	Independent variable: MVAIC	Independent variable: CEE	Independent variable: HCE	Independent variable: SCE	Independent variable: RCE	Independent variable: RDE
Constant	0.170 (1.33)	0.046 (0.61)	0.225* (1.80)	0.154 (0.91)	0.115 (0.69)	0.131 (0.79)
X	0.017*** (6.74)	0.313*** (15.02)	0.018*** (7.08)	-0.009 (-1.58)	0.054** (2.22)	0.049* (1.98)
SIZE	-0.008 (-1.11)	0.005 (1.29)	-0.010 (-1.42)	0.005 (0.58)	0.007 (0.81)	0.006 (0.66)
LEV	-0.129*** (-2.85)	-0.194*** (-7.40)	-0.119** (-2.67)	-0.201*** (-3.46)	-0.206*** (-3.62)	-0.204*** (-3.56)
OWN	0.023* (1.72)	-0.006 (-0.71)	0.022 (1.66)	0.001 (0.07)	0.002 (0.12)	0.0002 (0.01)
AGE	0.010 (0.54)	-0.027** (-2.52)	0.011 (0.60)	-0.043* (-1.85)	-0.048** (-2.06)	-0.044* (-1.89)
SALES	0.008* (1.80)	-0.016*** (-4.94)	0.008* (1.84)	0.009 (1.56)	0.010* (1.74)	0.010* (1.76)
YEAR	Yes	Yes	Yes	Yes	Yes	Yes
N	62	62	62	62	62	62
Adj. R^2^	0.5433	0.8376	0.5638	0.1963	0.2296	0.2159
F	11.37***	45.94***	12.27***	3.13***	3.60***	3.40***

Notes: * *p* < 0.10

** *p* < 0.05

*** *p* < 0.01. *t*-values are in parentheses.

**Table 8:** Regression results of Models (1)–(6) during COVID-19.

Variable	Model (1)	Model (2)	Model (3)	Model (4)	Model (5)	Model (6)
	Independent variable: MVAIC	Independent variable: CEE	Independent variable: HCE	Independent variable: SCE	Independent variable: RCE	Independent variable: RDE
Constant	0.094 (1.15)	0.067 (0.83)	0.137* (1.67)	0.004 (0.003)	-0.002 (-0.02)	-0.004 (-0.04)
X	0.014*** (7.93)	0.113*** (8.19)	0.016*** (8.21)	-0.003 (-0.78)	0.015 (0.96)	0.001 (0.06)
SIZE	-0.001 (-0.32)	0.005 (1.30)	-0.003 (-0.73)	0.010* (1.82)	0.010* (1.85)	0.010* (1.84)
LEV	-0.178*** (-7.02)	-0.180*** (-7.19)	-0.172*** (-6.81)	-0.227*** (-7.24)	-0.226*** (-7.18)	-0.230*** (-7.32)
OWN	0.030*** (3.17)	0.006 (0.68)	0.029*** (3.14)	0.010 (0.89)	0.011 (0.94)	0.012 (1.00)
AGE	0.001 (0.12)	-0.028** (-2.40)	0.004 (0.30)	-0.024 (-1.59)	-0.024 (-1.62)	-0.023 (-1.51)
SALES	0.042*** (3.14)	0.061*** (5.00)	0.038*** (2.84)	0.098*** (6.77)	0.098*** (6.74)	0.098*** (6.77)
YEAR	Yes	Yes	Yes	Yes	Yes	Yes
N	109	109	109	109	109	109
Adj. R^2^	0.7250	0.7318	0.7323	0.5564	0.5578	0.5538
F	41.67***	43.09***	43.20***	20.36***	20.46***	20.15***

Notes: * *p* < 0.10

** *p* < 0.05

*** *p* < 0.01. *t*-values are in parentheses.

Next, this study examines whether the impact of IC and its elements on firms’ financial performance is different for companies with high leverage and companies with low leverage based on the median value of LEV. The results are shown in Tables 9 and 10. The impact of IC and HC on ROA is greater in low leveraged companies. In addition, for highly leveraged companies, SC has a negative impact on ROA, and RC has a positive impact. However, the impact of SC and RC is not significant for low leveraged companies. RDE has no significant impact on both types of companies.

**Table 9:** Regression results of Models (1)–(6) for companies with high leverage.

Variable	Model (1)	Model (2)	Model (3)	Model (4)	Model (5)	Model (6)
	Independent variable: MVAIC	Independent variable: CEE	Independent variable: HCE	Independent variable: SCE	Independent variable: RCE	Independent variable: RDE
Constant	0.156 (1.51)	0.093 (1.07)	0.220** (2.18)	0.199 (1.34)	0.175 (1.19)	0.179 (1.20)
X	0.014*** (9.11)	0.145*** (12.23)	0.016*** (9.61)	-0.008* (-1.76)	0.040* (1.96)	0.021 (1.48)
SIZE	-0.004 (-0.83)	0.003 (0.81)	-0.007 (-1.38)	0.006 (0.87)	0.006 (0.95)	0.007 (0.96)
LEV	-0.276*** (-4.64)	-0.161*** (-3.10)	-0.290*** (-5.04)	-0.359*** (-4.28)	-0.347*** (-4.17)	-0.364*** (-4.32)
OWN	0.034*** (3.73)	0.009 (1.20)	0.036*** (3.99)	0.022 (1.67)	0.022* (1.68)	0.021 (1.62)
AGE	0.023 (1.39)	-0.029** (-2.26)	0.028* (1.77)	-0.034 (-1.57)	-0.035 (-1.63)	-0.033 (-1.54)
SALES	0.009** (2.19)	-0.001 (-0.35)	0.009** (2.30)	0.015** (2.66)	0.015*** (2.76)	0.016*** (2.75)
YEAR	Yes	Yes	Yes	Yes	Yes	Yes
N	85	85	85	85	85	85
Adj. R^2^	0.6313	0.7409	0.6519	0.2545	0.2616	0.2457
F	16.98***	27.69***	18.48***	4.19***	4.31***	4.04***

Notes: * *p* < 0.10

** *p* < 0.05

*** *p* < 0.01. *t*-values are in parentheses.

**Table 10:** Regression results of Models (1)–(6) for companies with low leverage.

Variable	Model (1)	Model (2)	Model (3)	Model (4)	Model (5)	Model (6)
	Independent variable: MVAIC	Independent variable: CEE	Independent variable: HCE	Independent variable: SCE	Independent variable: RCE	Independent variable: RDE
Constant	0.314*** (2.89)	0.037 (0.35)	0.386*** (3.49)	-0.049 (-0.33)	-0.060 (-0.40)	-0.045 (-0.30)
X	0.025*** (9.35)	0.267*** (8.58)	0.026*** (9.52)	-0.001 (-0.25)	0.017 (0.81)	-0.008 (-0.61)
SIZE	-0.016** (-2.65)	0.007 (1.18)	-0.018*** (-2.96)	0.012 (1.57)	0.013 (1.66)	0.012 (1.50)
LEV	-0.116*** (-3.18)	-0.218*** (-5.88)	-0.087** (-2.35)	-0.201*** (-3.87)	-0.200*** (-3.87)	-0.201*** (-3.88)
OWN	0.039*** (2.85)	-0.010 (-0.76)	0.031** (2.34)	-0.014 (-0.78)	-0.014 (-0.76)	-0.014 (-0.75)
AGE	0.004 (0.35)	-0.030** (-2.56)	0.001 (0.04)	-0.032* (-1.87)	-0.034* (-1.98)	-0.030* (-1.76)
SALES	0.029** (2.13)	0.048*** (3.49)	0.022 (1.57)	0.078*** (4.18)	0.077*** (4.13)	0.078*** (4.23)
YEAR	Yes	Yes	Yes	Yes	Yes	Yes
N	86	86	86	86	86	86
Adj. R^2^	0.6873	0.6586	0.6935	0.3285	0.3337	0.3312
F	21.76***	19.22***	22.37***	5.62***	5.73***	5.68***

Notes: * *p* < 0.10

** *p* < 0.05

*** *p* < 0.01. *t*-values are in parentheses.

### 4.5. Additional analysis

This study further examines the nonlinear relationship between IC and its components and firm financial performance. Table 11 shows an inverted-U relationship between IC and HC and ROA and a U-shaped relationship between physical capital and ROA. It can be inferred that companies need to reasonably allocate their internal IC resources in order to maximize the efficiency of value generation. Ecological protection and environmental governance sector belongs to the tertiary industry, and sufficient physical assets provide the basis for the operation of such companies.

**Table 11:** Regression results of Models (1)–(6).

Variable	Model (1)	Model (2)	Model (3)	Model (4)	Model (5)	Model (6)
	Independent variable: MVAIC	Independent variable: CEE	Independent variable: HCE	Independent variable: SCE	Independent variable: RCE	Independent variable: RDE
Constant	0.132** (2.14)	0.047 (0.71)	0.201*** (3.70)	0.020 (0.21)	0.020 (0.21)	0.018 (0.19)
X	0.023*** (15.12)	0.198*** (11.95)	0.028*** (19.00)	0.006 (1.13)	0.003 (0.16)	0.015 (1.45)
X^2^	-0.001*** (-6.53)	0.032*** (3.24)	-0.002*** (-9.71)	-0.002** (-2.62)	-0.021* (-1.74)	-0.013*** (-3.22)
SIZE	-0.004 (-1.27)	0.007** (1.98)	-0.008** (-2.58)	0.011** (2.13)	0.011** (2.14)	0.011** (2.19)
LEV	-0.119*** (-6.07)	-0.184*** (-8.91)	-0.093*** (-5.33)	-0.202*** (-6.97)	-0.204*** (-7.01)	-0.203*** (-7.10)
OWN	0.018** (2.54)	0.003 (0.38)	0.014** (2.31)	0.005 (0.46)	0.004 (0.34)	0.003 (0.34)
AGE	-0.004 (-0.47)	-0.038*** (-4.15)	-0.002 (-0.22)	-0.043*** (-3.28)	-0.044*** (-3.33)	-0.043*** (-3.31)
SALES	0.010*** (2.85)	-0.002 (-0.61)	0.008*** (2.80)	0.018*** (3.37)	0.018*** (3.39)	0.018*** (3.39)
YEAR	Yes	Yes	Yes	Yes	Yes	Yes
N	171	171	171	171	171	171
Adj. R^2^	0.7355	0.6851	0.7965	0.3617	0.3558	0.3688
F	68.52***	53.82***	96.04***	14.76***	14.41***	15.19***

Notes: * *p* < 0.10

** *p* < 0.05

*** *p* < 0.01. *t*-values are in parentheses.

## 5. Discussion

IC stands as a pivotal factor in promoting the healthy and long-term development of companies, and its role has attracted much attention in emerging markets. Furthermore, the advent of digital transformation has revolutionized the way firms bridge knowledge gaps and facilitated enhanced knowledge acquisition, which bolsters overall firm performance. Our research delves into the independent and joint effects of IC and digital transformation on firm financial performance in China’s ecological protection and environmental governance sector. It can guide companies in enhancing their knowledge management practices and improving the value creation efficiency of IC in the digital era.

The study shows that to some extent IC enhances financial performance in China’s ecological protection and environmental governance sector. Beyond a point, it will lead to a decline in firm financial performance. This corroborates the findings of Xu and Zhang [51] and Xu et al. [78]. China’s environmental protection and ecological governance industry is at the early stage of development, and the accumulation of IC (including knowledge, technology, patents, etc.) has a significant positive impact on corporate performance. By investing in new technologies, optimizing governance structure and enhancing environmental awareness, such companies can rapidly enhance market competitiveness and achieve business growth. However, high levels of IC investments can lead to substantial R&D costs and human resource costs. When the growth rate of these costs exceeds that of revenue, the overall performance of these enterprise may be affected. Although IC is regarded as a unique resource, companies still need to effectively harness it. During COVID-19, IC also plays a prominent role in improving financial performance, which supports the findings of Xu et al. [43]. The impact of IC in low leveraged companies is greater, which suggests that ecological protection and environmental governance companies should maintain a good capital structure to ensure the efficiency of IC.

Regarding IC components, HC has an inverted-U relationship with financial performance. Excessive investment in HC will increase expenditure on employees, thus reducing firm performance. RC is found to have a positive impact on financial performance, while structural and innovation capitals have no significant impact. For ecological protection and environmental governance companies, their main customers are government departments and enterprises, and close ties with them can ensure revenue. In the digital era, if companies do not adjust their organizational structure to adapt to the digital environment, it will lead to low operational efficiency, thus reducing firm profitability. R&D activities require continuous investment [62, 79], the insufficient investment in R&D might lead to the insignificant impact of innovation capital. During COVID-19, the impact of RC is no longer significant. The economic impact of COVID-19 pandemic is severe, and many businesses face declining revenues and rising costs [43]. In such circumstances, businesses need to prioritize cost reduction, rather than developing and maintaining RC. Additionally, RC is instrumental in gaining new resources and technologies, thus fostering corporate innovation [80]. However, Ashraf et al. [81] pointed out that RC positively affects the profitability of hospitality firms before and during COVID-19. This study suggests that companies should continue to invest in RC in the post-COVID-19 era. In addition, we find that SC and RC has an impact on financial performance of highly leveraged companies. Companies with high asset-liability ratio face great financial pressures, which constrains their operational and investment flexibility. If SC of these companies is not able to rapidly adapt to market changes, this constraint will be further exacerbated, thereby impacting financial performance. Highly leveraged companies often require more external financing to support their operations and expansion, and long-term and stable RC can provide them with more convenient financing channels and lower financing costs, thereby influencing financial performance.

Surprisingly, the results show that digital transformation has a negative impact on firm financial performance. This could be explained by the fact that the early stage of digital transformation requires huge investment, including purchasing new hardware and software, training employees, and integrating systems. Ecological protection and environmental governance companies are in the early stages of development, and these costs become a burden in the short term. This finding is contrary to most previous studies [13, 15, 16, 60, 70], while Nguyen-Thi-Huong et al. [82] and Yang and Masron [83] found the same results with the data of banks.

In addition, it is found that digital transformation strengthens the positive relationship between IC and its two elements (HC and RC) and firm financial performance. Digital transformation can improve employee skills and knowledge through online training and promote team collaboration by using instant messaging tools and online collaboration platforms, and it also can enhance customer interaction through social media and optimize supply chain management through data analysis. Digital transformation does not moderate the relationship between SC and financial performance. This might be because digital transformation not only involves technological changes, but also requires corresponding adjustments to organizational structure and processes. At present, ecological protection and environmental governance companies do not have a flexible structure to quickly adapt to the changes brought by digital transformation. The insignificant moderating effect in the relationship between innovation capital and firm performance could be that corporate digital transformation processes are not closely integrated with R&D processes.

## 6. Conclusion

This paper aims to investigate the impact of IC on financial performance of publicly traded companies in China’s ecological protection and environmental governance sector. It also examines whether the impact of IC and its elements on firms’ financial performance is different before and during COVID-19 and between highly leveraged companies and low leveraged companies. In addition, the moderating role of digital transformation is examined. The MVAIC model is used to measure IC, and text analysis is used to measure the level of digital transformation. The main conclusions can be listed as follows. First, IC, HC, and RC are the contributors to firm financial performance measured by ROA. The relationship between RC and innovation capital and financial performance is positive before COVID-19, and it is not significant during COVID-19. For highly leveraged companies, there is a negative relationship between SC and ROA and a positive relationship between RC and ROA, while these impacts are not significant for low leveraged companies. Second, digital transformation strengthens the positive relationship between IC and two components (HC and RC) and firm financial performance. Third, there is a nonlinear relationship between IC and firm financial performance.

The theoretical contributions of this paper can be summarized in three aspects. First, this study enriches the literature by deepening the understanding of the value creation of IC in the digital environment. Specifically, we analyze how four IC components affect firm performance in the process of corporate digital transformation. In addition, this study can help managers understand the role of IC and its components in times of crisis. Secondly, this study uncovers the moderating mechanism of corporate digital transformation in the relationship between IC and firm financial performance. Finally, this study helps companies find new channels to improve their performance through reasonable management of IC in their digital transformation.

The practical implications are put forward as follows. First, managers in ecological protection and environmental governance sector should continually invest in intellectual assets and improve employees’ digital literacy. They should organize regular training sessions and workshops to equip employees with the latest digital tools and technologies relevant to their field and encourage employees to pursue online courses and certifications in environmental informatics and data analysis [84]. Meanwhile, they need to be concerned about the role of SC and innovation capital as they are beneficial to business transformation in the digital era. Second, these companies should fully attach importance to the crucial role of HC in the process of corporate digital transformation and increase the cultivation of digital talent. Third, they should deepen the integration of digital technology and enterprise innovation processes and enhance corporate innovation capabilities. Fourth, companies should leverage the information advantages of digital transformation to establish long-term and stable relationships with suppliers and customers, thus enhancing sustainable competitive advantage. Finally, the governmental departments should issue relevant policies to encourage such companies to invest in IC and accelerate digital transformation for the booming of new business models.

This study has some limitations that should be addressed. First, our study is relatively small and limited to ecological protection and environmental governance industry, and further research could extend to other industries. Ecological protection and environmental governance industry is an emerging industry with upgraded technology, while traditional industries such as manufacturing industry might rely more on resources, which leads to the different impacts of IC on firm performance. A future investigation on how IC can affect firm performance in different industries is necessary. Second, although the current literature widely adopts the MVAIC method as a measurement of IC, other IC elements (e.g. process capital) could be included in future studies. Third, some external factors that might influence IC are not taken into account in this study, and further research could add these variables into the research models. In addition, other methods of digital transformation measurement could be used in future studies.

## Supporting information

S1 Data (XLS)
